# Prediction and analysis of analytical ultracentrifugation experiments for heterogeneous macromolecules and nanoparticles based on Brownian dynamics simulation

**DOI:** 10.1007/s00249-018-1322-2

**Published:** 2018-07-20

**Authors:** J. García de la Torre, J. G. Hernández Cifre, A. I. Díez Peña

**Affiliations:** Department of Physical Chemistry, University of Murcia, 30071 Murcia, Spain

**Keywords:** Analytical ultracentrifugation, Brownian dynamics, Diffusion, Sedimentation

## Abstract

In the prediction of sedimentation profiles in analytical ultracentrifugation, the counterflow due to diffusion must be taken into account for a proper analysis of experimental data in the determination of molecular properties. This is usually achieved by numerical solution of the Lamm equation. This paper presents an alternative approach, in which the displacement of the solute in the cell, resulting from the opposite effects of ultracentrifugal force and diffusional drift, is described by Brownian dynamics simulation of the solute particles. The formalism is developed for heterogeneous solutes, composed of several species, and implemented in computational schemes and tools. The accuracy of the procedure is verified by comparison with other methods based on the Lamm equation, and its efficiency is illustrated. The possibilities offered by the Brownian dynamics methods in the determination of solute properties and sample composition are demonstrated.

**Electronic supplementary material:**

The online version of this article (10.1007/s00249-018-1322-2) contains supplementary material, which is available to authorized users.

## Introduction

Analytical ultracentrifugation (AUC) is a classical (Svedberg and Rinde 1924), yet contemporary (Uchimaya et al. 2016) technique for the characterization of macromolecular and colloidal particles in solution. As described in numerous recent reviews, book chapters and monographs (see, for instance, Uchimaya et al. 2016; Schuck et al. 2016; Patel et al. 2016), AUC permits the separation of the components of a solute sample in the centrifugal field, as well as the characterization of their individual solution properties and, even, their interactions.

The prediction of the time course of sedimentation is required not just for the simulation of experiments from the properties of the solute, but also for the inverse problem, i.e., the analysis of the outcome of the sedimentation run for the determination of the molecular properties. If the motion caused by the centrifugal field was the only contribution to solute transport, the mathematical description of sedimentation would be very easy. However, there is another effect to be considered: the diffusional counterflow caused by the concentration gradient that evolves as the solute is concentrating toward the bottom of the cell. The contribution of diffusion could be neglected when sedimentation is overwhelmingly fast, as could be the case when particles are sufficiently large and/or rotor speed is sufficiently high. Indeed, some treatments of AUC neglect diffusion to concentrate on other relevant aspects. However, in many practical instances, this is not the case; the effect of diffusion must be considered for a rigorous description of AUC experiments. Indeed, rather than complicating matters, the consideration of diffusion in the analysis of AUC data provides additional valuable molecular information.

As described below, the sedimentation–diffusion balance has been customarily expressed by means of the Lamm equation, which combines the flux of the bulk transport caused by the centrifugal force, associated with the bulk sedimentation velocity, with the flux of diffusion, which is represented by Fick’s law. In the proposal that we put forward (Díez et al. 2011), instead of the macroscopic view of diffusion, we adopt a microscopic view described by the fundamental Einstein laws of Brownian motion (Einstein 1905). The replacement of the Fickian by the Einstenian description of diffusion, with the solute represented by discrete particles, replaces the Lamm equation by a Brownian dynamics algorithm that is remarkably simple, computationally efficient, and adaptable to the arbitrarily complex situations that are often found in AUC experiments. Although AUC experiments are essentially macroscopic, the paradigm of computer simulation using particles is certainly applicable and effective (Hockney and Eastwood 1988).

In our previous paper Díez et al. (2011), we proposed this novel approach based on Brownian dynamics (BD) simulation and, as proofs of concept, we presented some simple test examples for monocomponent systems. In the present paper, we develop the methodology further and present various applications of practical importance. In addition to improving and benchmarking the new computational procedures, we consider in detail the case of heterogeneous solutes. In our scheme, heterogeneity is considered not only as a polydispersity of molecular weight, but also in molecular composition, as happens with mixtures of particles with different densities.

The present developments are implemented in a suite of computer programs, SimuSed, which currently comprises a program, PrediSed, for BD-based prediction of sedimentation profiles and another, AnaSed, which carries out the analysis of these profiles in the determination of their sedimentation coefficients, other molecular information, and sample composition.

## Theoretical framework

### Basic aspects of analytical ultracentrifugation

Under the action of a centrifugal force, due to rotation with angular velocity $\omega$ at a distance *r* of the rotation axis, a particle experiences a force $m_\mathrm{b} \omega ^2 r$, where $m_\mathrm{b}=m(1-\bar{v} \rho )$ is the buoyant mass, and *m* is the particle mass, $\bar{v}$ its specific volume, and $\rho$ the density of the solution in which is immersed. The motion of the particle, with velocity *v*, is opposed by a frictional force $-v f$, where *f* is the friction coefficient of the particle in the viscous solution. The sedimentation coefficient is defined as the ratio of the linear velocity to the centrifugal acceleration, $s=v/(\omega ^2 r)$, so

1 $$\begin{aligned} v = \frac{\mathrm{d}r(t)}{\mathrm{d}t} = s \omega ^2 r(t). \end{aligned}$$

The balance of the centrifugal and frictional force gives

2 $$\begin{aligned} s = \frac{m_{\mathrm{b}}}{f} = \frac{M (1-\bar{v} \rho )}{N_\mathrm{A} f} = \frac{M^{(\mathrm{b})}}{N_A f}. \end{aligned}$$

Here, *M* is the molecular weight of the particle, $N_\mathrm{A}$ is Avogadro’s number, and $M^{(\mathrm{b})} =M (1-\bar{v} \rho )$ is a buoyancy-corrected molecular weight. According to the theory for diffusion in solution, *f* is related to the diffusion coefficient by the Einstein equation, $D = k_\mathrm{B}T/f$, where $k_\mathrm{B}$ is Boltzmann constant and *T* the absolute temperature. Combined with Eq. (2), they give the Svedberg equation:

3 $$\begin{aligned} \frac{s}{D} = \frac{M (1-\bar{v} \rho )}{RT} = \frac{M^{(\mathrm{b})}}{RT}, \end{aligned}$$

where *R* is the perfect gas constant. As described in textbooks (van Holde et al. 1998; Sun 2004; Hiemenz and Lodge 2007; Serdyuk et al. 2007), Eqs. (1)–(3) provide a basic description of the sedimentation experiment, from which the sedimentation coefficient and the other properties involved can be determined.

If the particle motion was determined just by this description, according to Eq. (1), the equation of motion would be trivially

4 $$\begin{aligned} r(t')=r(t)\exp \left[ s \omega ^2 (t'-t) \right] . \end{aligned}$$

By applying Eq. (4) to the particles of the sedimentation boundary, the well-known expression for estimating the sedimentation coefficient from the time-dependent position of the boundary is obtained:

5 $$\begin{aligned} \ln \left[r(t_0+\tau )/r(t_0) \right] = s \omega ^2 \tau , \end{aligned}$$

where $r(t_0)$ is some initial position of the boundary, which is located at $r(t_0+\tau )$ after a time $\tau$ has elapsed. The sedimentation coefficient could be determined from the slope of a plot of the term in the right-hand side of Eq. (5) vs. $\tau$.

**Fig. 1:**
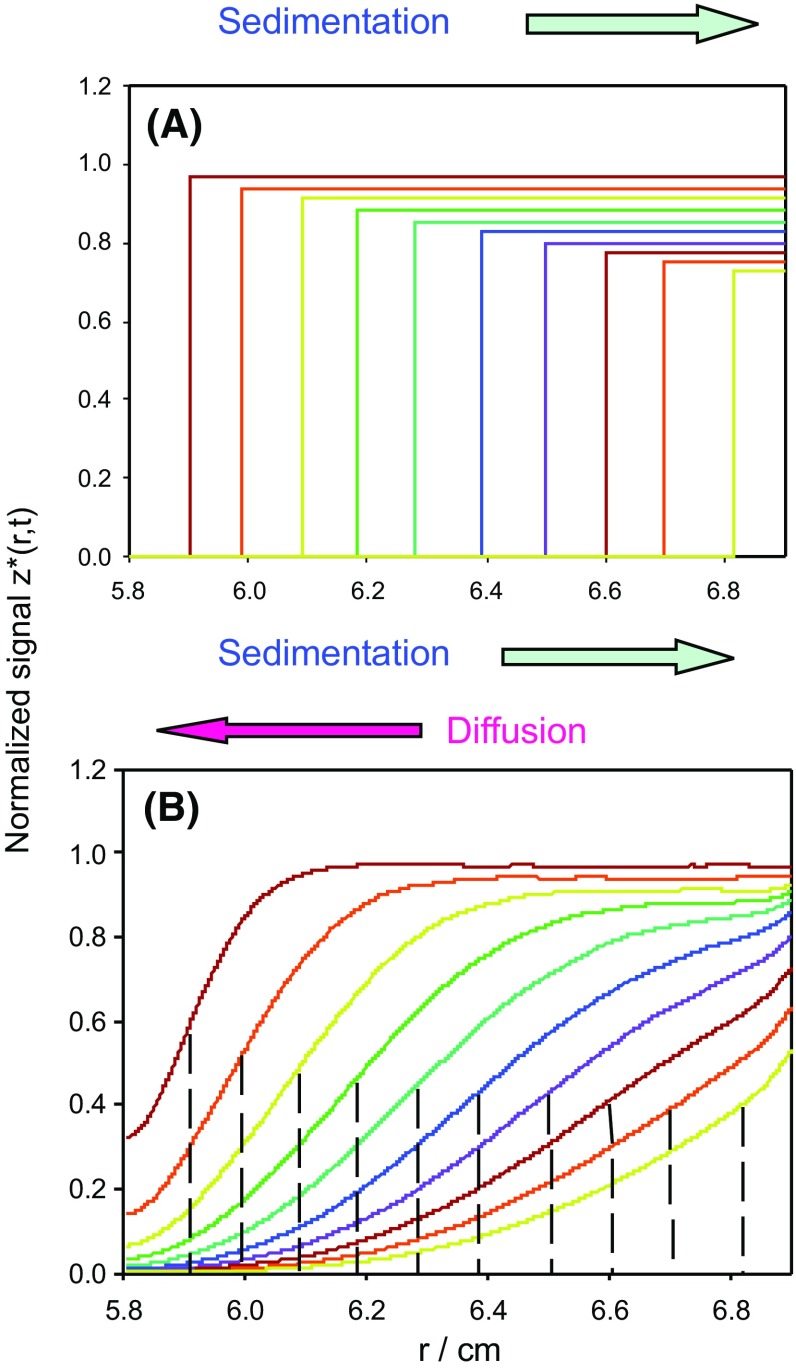
Concentration profiles, *c*(*r*, *t*) vs. *r*, during sedimentation of lysozyme in water at 20 $^{\circ }$C ($s= 1.80$ S, $M = 14.3$ kDa, $\bar{v} = 0.703$ cm$^3$/s, $\omega = 40{,}000$ rpm) during 6 h at intervals of 80 min. **a** Hypothetical profiles calculated neglecting diffusion. **b** Real profiles, predicted by Brownian dynamics simulation, as implemented in PrediSed with $N_\mathrm{part}=10^7$ particles and $N_\mathrm{s} = 50$ time steps

If the displacement caused by the centrifugal field was the only contribution to the motion of the solute molecules, the sedimentation boundaries would be sharp steps, as indicated in Fig. 1a. However, this cannot be the only contribution; actually, the concentration gradient created by the migration of particles in one direction causes a diffusive motion in the opposite direction. This effect is observed as a spread of the sedimentation boundary (as illustrated in Fig. 1b), whose analysis for the determination of *s* becomes more complex.

In the traditional AUC theory, diffusion is described by a macroscopic flux expressed by Fick’s law and determined by the diffusion coefficient, *D*. The net flux is the balance of the centrifugal and diffusional fluxes:

6 $$\begin{aligned} J(r,t) = s \omega ^2 r \cdot c(r,t) - D \frac{\partial c(r,t)}{\partial r}, \end{aligned}$$

which along with the condition of mass, $\partial c(r,t) / \partial t = -\,\partial J(r,t) / \partial r$, determines the dependence of the particle concentration on time and position, *c*(*r*, *t*), governed by the Lamm equation:

7 $$\begin{aligned} \frac{\partial c}{\partial t} = D \left[ \frac{\partial ^2 c}{\partial r^2} + \frac{1}{r}\frac{\partial c}{\partial r}\right] - s \omega ^2 \left[ r \frac{\partial c}{\partial r} + 2c \right] . \end{aligned}$$

In this form, the Lamm equation is written in cylindrical coordinates, as required by the radial geometry of the AUC setup, with a sector-shaped geometry of the cell. The solution of the differential equation of Lamm (which is quite difficult even in monodimensional form, if one neglects the radial geometry) is further complicated by the two bounds imposed by the meniscus of the solution and the bottom of the cell, at which the net flux must be zero at any time.

A clever scheme for the numerical, finite-element solution of the Lamm equation was presented by Claverie et al. (1975). The complexity of the procedure (as well as the size of the data set provided by modern ultracentrifuges) precluded an operational implementation of their method until c.a. 2000 (Demeler and Saber 1998; Schuck 1998; Stafford and Sherwood 2004) and it is now at the core of the existing software packages for the AUC analysis (Demler et al. 2017; Schuck et al. 2017; Stafford et al. 2017).

### BD simulation algorithm

In our previous paper Díez et al. (2011), we proposed that, instead of a macroscopic, Fickean treatment of diffusion, the diffusive component of the particle motion could be considered microscopically in terms of the fundamental equations of Brownian motion, so the time course of sedimentation can be described by a Brownian dynamics algorithm, which is very simple, general, and computationally efficient. Here, we summarize the basis aspects of our algorithm for the case of a single solute, which will, afterwards, be generalized to the case of a heterogeneous solute.

As in the macroscopic description, the trajectory of a solute particle is a superposition of displacements caused by simultaneous sedimentation and diffusion. The first component is the deterministic drift due to the centrifugal force, governed by the sedimentation velocity equation, Eq. (1), which, in integrated form, can be written as Eq. (4), so the displacement due to the centrifugal field during time $\Delta t$ would be

8 $$\begin{aligned} \Delta r_\mathrm{sed} = r(t) \left[ 1- \exp (s \omega ^2 \Delta t )\right] . \end{aligned}$$

The second displacement is that due to Brownian motion; according to the Einstein microscopic theory of diffusion, it has a random value with Gaussian distribution of zero mean and variance:

9 $$\begin{aligned} \langle (\Delta r_\mathrm{brow})^2 \rangle =2 D \Delta t, \end{aligned}$$

so the final position after the time step would be

10 $$\begin{aligned} r(t+\Delta t) = r(t) + \Delta r_\mathrm{sed} + \Delta r_\mathrm{brow}. \end{aligned}$$

Equation (8) is obviously valid for an arbitrary time step, $\Delta t$. On the other hand, thanks to the fractal nature of Brownian motion, the Brownian steps follow Eq. (9) regardless of how long the step is. Therefore, in an unbounded system, Eq. (10) is valid for arbitrary long time steps. The exceptions come from the bounds imposed by the solution meniscus and the cell bottom. In our previous paper, we devised ad hoc protocols to handle these exceptions, and to make them less frequent, we proposed dividing the duration of the sedimentation experiment in a sufficient number of time steps. We noticed that only 50–100 steps suffice, introducing quite small disturbances in the predictions, and only at the extremes of the solution. Actually, these extremes are affected by other instrumental effects and are usually disregarded in the analysis of experimental data.

Thus, the trajectory of one sedimenting particle can be simulated with the simple algorithm described by Eqs. (8)–(10). The duration of the experiment, $t_\mathrm{run}$, is discretized into $N_{\mathrm{s}}$ intervals of duration $\tau =t_\mathrm{run}/N_\mathrm{s}$, so the *j*th instant corresponds to time $j\tau$. In addition, the position in the solution is divided into $N_r$ sections of width $\chi =(r_\mathrm{b}-r_\mathrm{m})/N_r$; the midpoint of the *i*th interval is $r_i = r_\mathrm{b} +(i- \frac{1}{2})\chi$. We note that, in the simulation, *r* is a continuous variable; from Eqs. (8)–(10), it can take any value between $r_\mathrm{m}$ an $r_\mathrm{b}$. It is discretized in $N_r$ partitions just to evaluate the signal $z(r_i,t_j)$ from the number of particles, as indicated below.

The solute is represented in the simulation by a large number of particles, $N_\mathrm{part}$. The trajectory of each particle is simulated, and at each instant *j*, the interval *i* where the particle is located is determined. Then, the *n*(*i*, *j*) counter, which gives the number of particles located at *i* at time *t*, is increased by one unit. The starting point $r_0$, where a particle departs, is chosen under the condition that the concentration is initially uniform through the sector-shaped cell. Considering that the volume of the intervals of width $\chi$ is proportional to *r*, we found (Díez et al. 2011; Díez 2014) that the condition of uniform initial concentration is fulfilled taking

11 $$\begin{aligned} r_0 = \sqrt{u({r_\mathrm{b}^2-r_\mathrm{m}^2})+r_\mathrm{b}^2}, \end{aligned}$$

where $u\in (0,1)$ is a uniformly distributed random number. At the end of the simulations, we obtain the particle counters for each position and time interval, *n*(*i*, *j*). Taking into account again the sector shape of the cell, which causes a radial dilution effect, it can be shown that the concentration at some interval is related to the fraction of the total particles as follows (Díez et al. 2011; Díez 2014):

12 $$\begin{aligned} \frac{c(r_i,t_j)}{c_0} = \frac{z(r_i,t_j)}{z_0} = \frac{(r_\mathrm{b}^2-r_\mathrm{m}^2)}{2\chi } \frac{1}{r_i} \frac{n(i,j)}{N_\mathrm{part}}. \end{aligned}$$

As the signal, *z*, detected in the ultracentrifuge is proportional to concentration, the signal at each position and time relative to the initially uniform value, $z(r_i,t_j)/z_0$, is also given by the right-hand side of Eq. (12).

### Sedimentation of a heterogeneous solute: description and BD simulation

We now present the generalization of our simulation scheme to a solute composed of an arbitrary number, $n_c$, of components. Before going into details of the BD simulation for such a heterogeneous system, we consider the forms for the relationships between the detector signal and the concentrations of the components. At any position *r* and time *t*, the signal is assumed to be additive on contributions of each component, $z(r,t) = \sum _k z_k(r,t)$, $k=1,\ldots ,n_c$ and, of course, for the initial uniform signal $z_0= \sum _k z_{0,k}$. Contributions to signal are proportional to concentration. In the most frequent detection modes, absorbance and interference, the *z*s are proportional to the *mass* concentrations, *c*s, so $z_k = q_k c_k$, with $q_k$ being a constant related to the nature of the *k*th component and instrumental data. We also envision the case when signals would be proportional to the *molar* concentrations $c_{M,k} = c_k/M_k$ (for instance for a polydisperse sample with an end-tagged fluorophore in fluorescence detection). In both cases, the signal–concentration relationship can be condensed into the form:

13 $$\begin{aligned} z_{0,k} = \frac{q_k c_{0,k}}{M_k^{\alpha _k}}, \end{aligned}$$

with either $\alpha _k = 0$ or $\alpha _k = 1$ for dependence on either mass or molar concentration, respectively. We can define a fraction contributed by each component to the total signal:

14 $$\begin{aligned} y_k = \frac{z_{0,k}}{z_0} = \frac{q_k c_{0,k}/M_k^{\alpha _k}}{\sum _{k=1}^{n_c} q_k c_{0,k}/M_k^{\alpha _k}}, \end{aligned}$$

so that $$\sum _{k=1}^{n_c} y_k =1$$.

It is noteworthy that if the $q_k$ for all the species were identical (as it would be the case for a mixture of oligo- or polymeric components), then $y_k$ would coincide with the weight fraction if the signal is proportional to mass concentration:

15 $$\begin{aligned} y_k = \frac{ c_{0,k}}{\sum _{k=1}^{n_c} c_{0,k}} = w_k \quad (\alpha _k=0) \end{aligned}$$

or it would be equal to the number fraction if the signal is proportional to molar mass:

16 $$\begin{aligned} y_k = \frac{ c_{0,k}/M_k}{\sum _{k=1}^{n_c} c_{0,k}/M_k} = x_k \quad (\alpha _k=1). \end{aligned}$$

Nonetheless, we continue to consider the general case, in which $y_k$ expresses the sample composition in terms of the fractional signal contribution of component *k*.

In the BD simulation of the heterogeneous systems of $n_c$, non-interacting components, each will be represented by a subset of $N_{\mathrm{part},k}$ particles for component *k*, and the trajectories will be generated independently, as described above. By applying Eq. (12) for each of the $n_c$ species, and invoking the additivity of the signal at every time and position, we have

17 $$\begin{aligned} z(r_i,t_j) = {z_0} \frac{(r_{\mathrm{b}}^2-r_{\mathrm{m}}^2)}{2\chi }\frac{1}{r_i} \sum _{k=1}^{n_c} y_k \frac{n_k(i,j)}{N_{\mathrm{part},k}}. \end{aligned}$$

As in the case of a single species, $N_{\mathrm{part},k}$ can be arbitrarily chosen. In the computer implementation of the algorithm, we shall give an appropriate value for the total number of particles, $N_\mathrm{part}$ (as this value determines the computing time; vide infra), which will be partitioned into $n_c$ groups, $N_\mathrm{part} = \sum _{k=1}^{n_c}N_{\mathrm{part},k}$. The remaining problem is to distribute this number of particles among the components. Our criterion is that $N_{\mathrm{part},k}$ should be related to the $z_k$ contribution, whose statistical noise, which contributes to the noise of the total signal, is proportional to $1/\sqrt{N_{\mathrm{part},k}}$ (vide infra). A lengthy derivation based on this criterion and omitted here (details can be found in Díez 2014) concludes that the fraction $\phi _k$ of particles should be related to the contribution of component *k* to the loading, initial signal, as follows:

18 $$\begin{aligned} \frac{N_{\mathrm{part},k}}{N_\mathrm{part}} \equiv \phi _k = \frac{z_{0,k}^{2/3}}{\sum _{k=1}^{n_c} z_{0,k}^{2/3}} = \frac{y_k^{2/3}}{\sum _{k=1}^{n_c} y_k^{2/3}}. \end{aligned}$$

We insist that choices of the number of particles, if they are sufficiently numerous, are not essential for the final result. Equation (18) provides a way of distributing a given $N_{\mathrm{part}}$ that optimized the statistical noise of the outcome from the BD simulation.

## Simulation of sedimentation experiments

### Method: program PrediSed

Based on our BD algorithm, we have written a computer program, PrediSed, to predict the outcome of a sedimentation experiment of multi-component samples. Instrumental data are rotor speed $\omega$, temperature *T*, duration $t_\mathrm{run}$, and position of meniscus $r_\mathrm{m}$, and bottom $r_\mathrm{b}$. Data pertaining to the simulation are the number of time and position intervals, $N_\mathrm{s}$ and $N_r$, and the total number of particles, $N_\mathrm{part}$. The data needed for each of the $n_c$ components are the sedimentation coefficient $s_k$ and the buoyant molecular weight, $M^\mathrm{b}_k$, along with their fractional contribution, $y_k$, to the initially uniform signal, $z_0$ (Eq. (14)). We recall that the diffusion coefficient is determined by this pair of values as follows:

19 $$\begin{aligned} D_k=RTs_k/M^{(\mathrm{b})}_k. \end{aligned}$$

The program assigns $N_{\mathrm{part},k}$ particles to each component according to Eq. (18), generating a Brownian trajectory for each of them. The distribution of particles along the cell at the times of successive scans, $n_k(i,j)$, is evaluated by tracking the trajectories. By doing this for each component and by adding their contributions according to Eq. (17), the sedimentation profiles *z*(*r*, *t*) are calculated and stored in computer files with various optional formats, including that of the output files of the Beckman XL/I ultracentrifuge.

### Computational details

Owing to the stochastic nature of the Brownian simulation algorithm, the simulated signal shows a random noise that depends on the size of the sample, thus obviously decreasing with increasing number of particles in the sample, $N_\mathrm{part}$. In addition to this dependence, intrinsic to the simulation of particle trajectories, the noise of the *z*(*r*, *t*) values is seen to depend also on the number of partitions or bins in radial position, $N_r$. The signal at a given radial position, *r*, is evaluated from the number of particles, *n*, found in an interval around *r* (Eq. (12)) of width $\chi =(r_\mathrm{b}-r_\mathrm{m})/N_r$; if $N_r$ is increased for a given $N_\mathrm{part}$, the value of *n* will be smaller, and, therefore, more noisy. Nonetheless, the noise in the simulated results can be reduced with smoothing procedures. We have found the Savitzky–Golay smoothing filter (Savitzky and Golay 1964; Press et al. 1986) particularly useful. Smooth *z*(*r*, *t*) vs. *r* or *z*(*r*, *t*) vs. *t* series allow for the calculation of the time and position derivatives, $\partial z / \partial t$ and $\partial z / \partial r$.

The simplicity of our BD-based simulation algorithm has the very welcome consequence of computing efficiency. In this regard, it is noteworthy that the time steps, $\Delta t$, in our BD algorithm may be arbitrarily long; in practice, they may have the same duration as the time interval between scans, $\tau$, so the number of steps in the simulation would be exactly equal to the number of scans, $N_{\mathrm{s}}$. Furthermore, we have been able to adapt our BD code for typical multi-core architecture, present today even in personal computers, by inserting OpenMP directives. Thus, in a processor allowing a number $N_\mathrm{threads}$ of parallel computing threads (usually one or two at each core), the trajectories of $N_\mathrm{threads}$ particles can be run simultaneously, one on each thread.

**Fig. 2:**
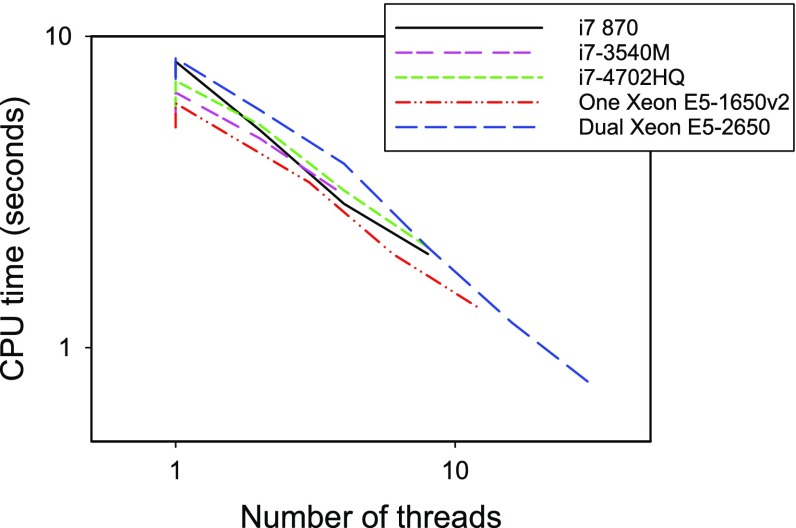
CPU times for a simulation of 10$^6$ particles, 100 time steps, 100 scans, run in personal computers and workstations (purchased 2014 or earlier) equipped with the indicated Intel i7 and Xeon processors. Values for varying numbers of parallel computing threads up to the maximum allowed by each processor

The gain in efficiency achieved by our parallel computing algorithm is illustrated in Fig. 2, which displays how CPU time is decreased by running multiple threads in various simple, inexpensive personal computers and workstations. The CPU times are for one full simulation using $N_\mathrm{part}=10^6$ particles and $N_\mathrm{s}=100$ scans, which would provide the above-mentioned low level of error, and would be typical settings for simulation (recall that CPU time is proportional to $N_\mathrm{part} \times N_\mathrm{s}$). We note how, in such simple platforms, the possibility of parallelizing the code of our algorithm can bring a tenfold increase in computing speed, so one full simulation can be made in less than 1 s of CPU time.

Thanks to the design, based on simulating trajectories of individual particles, and the great simplicity of the equations for particle motions, the computing code for the BD algorithm is particularly suitable for parallelization, thus taking full advantage of contemporary multi-core architectures. This has been demonstrated in this work with a conventional OpenMP implementation, but the algorithm is extremely well suited for the massively parallel architecture of graphical-processing-unit (GPU) processors. More advanced implementations of the BD algorithm and a detailed benchmarking of their computational performance are beyond the proof-of-concept scope of this paper.

**Fig. 3:**
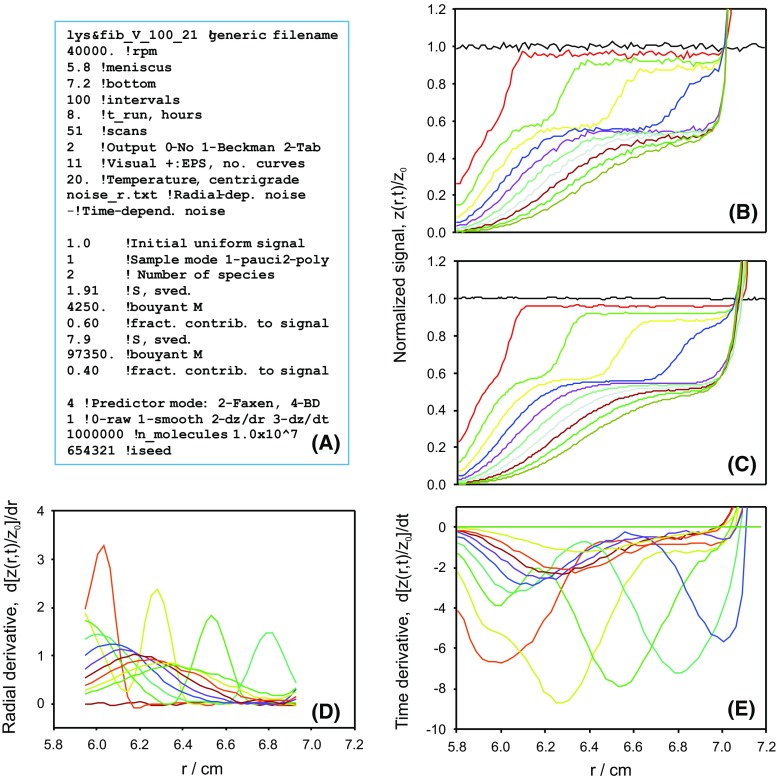
**a** Example of the input data file of PrediSed. This file corresponds to the conditions indicated in **a** . **b**–**e** Results from PrediSed, as displayed during program execution. **b** Raw results from the BD simulation. **c** Savitzky–Golay smoothed results. **d** Position derivative. **e** Time derivative

PrediSed has a single, quite simple input data file. An example is presented in Fig. 3. The primary data correspond to the instrumental setup, the sample, and options for calculation and presentation of results. The program includes a call to gnuplot (http://www.gnuplot.info) that allows us to visualize the resulting signal profiles during execution. In addition to the raw simulation results, smoothed profiles and time—or position—derivatives can be optionally presented. The time derivative $\mathrm{d}z(r,t)/\mathrm{d}t$ is particularly useful for analysis in the so-called “dcdt” mode (Stafford and Sherwood 2004; Stafford 1992; Philo 2000).

### Results

To illustrate the functioning and results of program PrediSed in the prediction of AUC experiments on heterogeneous system, we consider the case of a solution with two solutes of quite different solution properties. For solute 1, $s_1$ = 1.91 S and $M^{(\mathrm{b})}_1$ = 4250 Da, and for solute 2, $s_2$ = 7.9 S and $M^{(\mathrm{b}{} \mathbf )}_2 = 97,350$ Da (actually, these values correspond to lysozyme and fibrinogen, respectively). The fractional contributions are taken as $y_1=0.60$, $y_2=0.40$. We consider different experiments. One is a typical sedimentation velocity (SV) run for 8 h at 40,000 rpm. The other is an (approach to) sedimentation equilibrium (SE) experiment, at 10,000 rpm for 100 h, so that, by the end of the run, the equilibrium is fully reached.

**Fig. 4:**
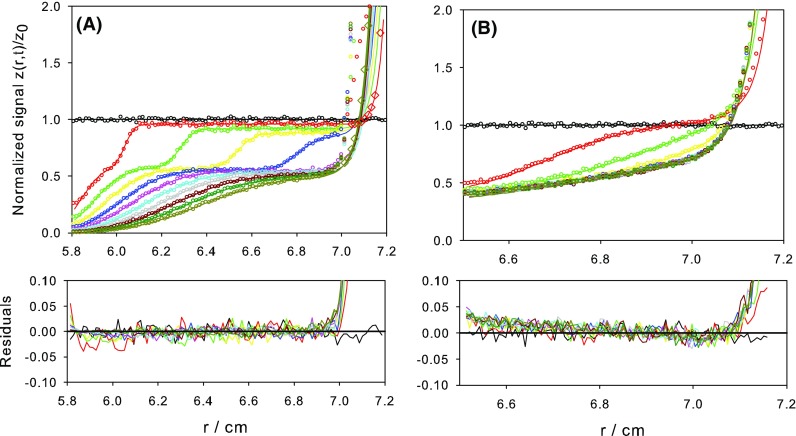
Results from PrediSed (circles and diamonds) compared with those from SEDFIT (lines). Two components, with $s_1$ = 1.91 S, $M^{(\mathrm{b})}_1$ = 4250 Da, $y_k=0.60$, $s_2$ = 7.9 S, $M^{(\mathrm{b})}_2$ = 97,350 Da, $y_2=0.40$. PrediSed simulations with $N_\mathrm{part}=10^6$ particles, $N_r=100$ radial intervals. 11 scans from $t=0$ (initially uniform signal) to $t_\mathrm{run}$. (A) SV run for $t_\mathrm{run}=$ 8 h at 40,000 rpm, $N_{\mathrm{s}}$ = 20 simulation steps or time intervals in PrediSed. Diamonds are PrediSed results with $N_\mathrm{s}$ = 200 for the scans at $t=$ 0.8 and 8 h. **b** SE run for $t_\mathrm{run}= 100$ h at 10,000 rpm, $N_s$ = 200 simulation steps or time intervals in PrediSed

To evaluate the performance of PrediSed, we compare its outcome with results simulated with the Generate> Single Non-interacting species mode of SEDFIT (version 15.01b of 2015) (Schuck et al. 2017). As indicated above, in our BD simulation algorithm, the time steps can be arbitrarily long without influencing the results except for terminal effects, which can affect the predicted concentrations at the meniscus and/or the bottom of the cell. The results in Fig. 4a for the SV experiment, from a simulation with $N_\mathrm{part} = 10^6$ and as few as $N_\mathrm{s}$ = 20 time steps, show that the agreement with the SEDFIT reference is fully acceptable, except at the bottom of the cell. For the most relevant purpose of simulating profiles, namely the interpretation of experimental data, this region of the cell, i.e., about 0.2 cm at the bottom, is usually disregarded in data analysis—the experimental data may, indeed, be faulty and the sharp concentration increase here may be of scarce significance. With such small $N_\mathrm{s}$, the CPU time is really small, about one-fifth of the values reported above for $N_\mathrm{s}=100$, while the computed results would be useful for data analysis.

In Fig. 4b, we present results for the SE experiment. In this case, we noticed that the PrediSed results are more sensitive to $N_\mathrm{s}$, with more severe deficiencies at the meniscus and bottom. More time steps are required for acceptable simulation results when compared to the SEDFIT reference. With $N_\mathrm{s}$ = 100, good agreement is achieved at the meniscus, and for $N_\mathrm{s}$ = 200, as shown in Fig. 4b, the PrediSed results are fully valid, with the exception of the terminal region at the bottom. As a rule of thumb, a general choice of $N_\mathbf s$ = 100 with the computing times reported above seems appropriate for any sedimentation conditions.

The primary source of uncertainty in the simulation results comes from the noise that depends on the size of the sample. This statistical noise, i.e., the uncertainty in the *z*(*r*, *t*) results, can be estimated by collecting values for repeated simulations in which the seed of the random number generator is varied; the standard deviation, $\delta z$, is a measure of the uncertainty. As indicated above, the uncertainty is found to decrease with increasing the number of particles, $N_\mathrm{part}$, and increase with decreasing the number of bins in the radial position, $N_r$. Detailed information on these dependencies is presented in Tables A1, A2, and A3 of the supplementary material given in Online Resource 1. The noise is found to be uniform throughout most of the cell, and nearly coincides with the deviations of the simulation results from the SEDFIT reference, thus indicating that the source of departure of the outcome of the BD scheme from that of the numerical solution of the Lamm equation is the simulation sample size. As shown by the first case in Table 1, the deviations in *z*(*r*, *t*) when $N_\mathrm{part}=10^6$, which seem unimportant for ordinary applications, can be further reduced with $N_\mathrm{part}=10^7$ to the order of $10^{-3} z_0$ (see Table A1 in Online Resource 1), i.e., comparable to the smallest experimental errors achievable with the most recent AUC instrumentation.

The other source of error, i.e., the systematic errors at the ends of the sample—mostly at the bottom of the cell—may be disregarded in the analysis of experimental data. However, we should note that they are simply consequence of the discretization of time and space in the BD simulation, and can be removed by decreasing the time steps, $\tau =t_\mathrm{run}/N_s$, i.e., by increasing the number of steps $N_\mathrm{s}$ for a given $t_\mathrm{run}$. Thus, with the typical displacement during one step (Eqs. (8) and (9)) being quite small, the occurrence of the bottom-hitting events would be very rare, as only the few molecules which are very close to the bottom could reach it. However, increasing $N_\mathrm{s}$ increases proportionally the computing time. In Fig. 4a, we intentionally display results for a extremely small $N_{\mathrm{s}}=21$, so the computation is very fast, but still gives valid results throughout most of the cell. As illustrated by the cases labeled as V/2 and E/2 in the supplementary material (Online Resource 1), the end effects near the bottom can be removed simply by increasing $N_\mathrm{s}$. In Fig. 4a, we have included PrediSed results in the bottom region, computed with $N_\mathrm{s}=201$ (CPU times just twice those in Fig. 2, about 2 s), showing that the agreement with the Lamm equation results from SEDFIT is fully satisfactory through the whole cell.

## Analysis of experimental data: program AnaSed

Lamm equation solvers and the BD simulation implemented in PrediSed provide calculations of the sedimentation profiles, $z(r,t;\{p\})$, for a given set of parameters $\{p\}$ pertaining to molecular properties of the components of the sample. The ultimate purpose of such calculations is the analysis of experimental data, intended to determine the molecular parameters $\{p\}$ by fitting the experimental profiles $z^{(\mathrm{e})}(r,t)$. Advanced methodology, based on Lamm equation solvers, already exists for that purpose (Demeler and Saber 1998; Schuck 1998; Stafford and Sherwood 2004; Demler et al. 2017; Schuck et al. 2017; Demeler and Gorbet 2016; Schuck 2016). To show how the BD calculation can be embedded into data analysis schemes, we have devised a simple tool, AnaSed, for data analysis.

We consider the possibility of a global, simultaneous analysis of various $n_\mathrm{exp}$ experiments, which may differ in some instrumental conditions, but have most other conditions in common. For each experiment, profiles $z_\mathrm{calc}^{(\mathrm{e})}$ are calculated, and the program aims to minimize the global square deviation between them and the experimental ones, $z_\mathrm{exp}^{(\mathrm{e})}$, by optimizing some set of parameters pertaining to the sample. In a heterogeneous system, these will include the essential quantities that govern the sedimentation of each component, $s_k$ and $M^{(\mathrm{b})}_k$ in our case, along with the sample composition (amount of each of them, in terms of either concentrations or contributions to signal, $y_k$), and any other parameter that could differ among experiments.

### Method

**Table 1:** Numerical results from the execution of AnaSed in the test case

$n_c$		$s_1$	$M^{(\mathrm{b})}_1$	$y_1$	$s_2$	$M^{(\mathrm{b})}_2$	$y_2$	$\Delta ^2$	Num. iter.
1	Initial	10.	20,000				1.000	0.13	
1	Best fit	3.11	3633				1.000	0.015	26
2	Initial	1.65	1816	0.500	6.22	7215	0.500	0.0030	
2	Best fit	1.90	4203	0.650	7.89	96250	0.350	0.0004	121
2	Exact	1.91	4250	0.650	7.90	97350	0.350		

The time–position-dependent signal for each experiment can be regarded as a function:

20 $$\begin{aligned} z^{(e)} \biggl [ (s, M^{(b)}, y)_k; (r_i, t_j)_e; (\omega , \ldots )_e \biggr ], \end{aligned}$$

For each experiment, there will be data for the positions and scans $(r_i, t_j)_e$ recorded, with eventually different values in the set $(\omega , \ldots )_e$, which includes the rotor speed along with other instrumental data (position of meniscus and bottom, etc). The determination of the best-fitting set of $n_c$ trios ($s_k , M^{(\mathrm{b})}_k, y_k)$, i.e., a set of $3n_c-1$ parameters (we recall that one of the $y_k$s is determined by $\sum _k y_k=1$) can be tackled using the conventional computational procedures for non-linear least squares. We intend to minimize a square deviation between calculated and experimental data, which is formulated as follows:

21 $$\begin{aligned} \Delta ^2 = \sum _{e=1}^{n_\mathrm{exp}} W_e \Delta _e^2, \end{aligned}$$

where $W_e$ is some statistical weight that could be assigned to each experiment ($\sum _e W_e =1$), and $\Delta _e^2$ is the square deviation between experimental and calculated $z^{(e)}$ for each experiment, which we formulate as

22 $$ \Delta _{e}^{2}  = \frac{1}{{N_{r} }}\frac{1}{{N_{{\text{s}}} }}\sum\limits_{{i = 1}}^{{N_{r} }} {\sum\limits_{{j = 1}}^{{N_{{\text{s}}} }} {\left[ {z_{{{\text{calc}}}}^{{(e)}} (r_{i} ,t_{j} ) - z_{{{\text{exp}}}}^{{(e)}} (r_{i} ,t_{j} )} \right]^{2} } } \bigg/ [z_{0}^{{(e)}} ]^{2}  $$

Note that the square deviation is averaged over all the $N_r\times N_\mathrm{s}$ data points and made relative to the initial signal to account for eventual differences in $z^{(e)}_{0}$ among experiments. Thus, the problem consists of the minimization of a function $\Delta ^2(p_1, p_2,\ldots )$, which depends non-linearly on a set of parameters to be optimized ${s_k , M^{(b)}_k, y_k}, k=1,\ldots ,n_c$. For the non-linear least-squares fitting, we have adopted the SIMPLEX algorithm of Nedler and Mead (1965), because, although it is not highly efficient, it was shown to yield acceptable results in all the cases that we tested. For the present development and proof-of-concept purposes, the profiles are generated with our PrediSed tool (recall that we have thoroughly verified that the PrediSed predictions are identical to those from SEDFIT). While the SIMPLEX method requires some initial estimation of the parameters, in a real situation, one would ignore not just their approximate values, but even the number of components. AnaSed adopts an heuristic, *ad hoc* approach. Initially, one single component is assumed, and a best fit of the set of data with only two parameters, $s_1$ and $M^{(\mathrm{b})}_1$ is carried out. Next, a two-component fit with five parameters is carried out; the initial values of $s_1$ and $M^{(\mathrm{b})}_1$ on one hand, and $s_2$ and $M^{(\mathrm{b})}_2$ on the other hand, are taken, respectively, as half and twice those resulting from the previous run, setting also $y_1=y_2=0.5$. In addition to reporting statistics of the fitting procedures, AnaSed (like PrediSed) also provides run-time visualization of the fits, so the quality of the fit can be readily appreciated. The numerical results from this example are shown in Table 1. After the first trial, with only one component, the second trial with two components converges very precisely to the correct values of the five parameters—i.e., those used in the generation of the “synthetic experimental” data. The agreement is excellent for both the high-speed and the low-speed experiments: the initial and fitted *z*(*r*, *t*) superimpose neatly (plots not shown, as they look the same as those in Fig. 4). The sum of square residuals, $\Delta ^2=4\times 10^{-5}$, amounts to a relative rms (root-mean-square) deviation of just 0.7%, which is just what comes from the noise in the initial data. The whole analysis requires a CPU time of about 1 min in a conventional personal computer.

## Concluding remarks

In our previous publication Díez et al. (2011), we put forward the possibility of solving AUC problems from a microscopic approach, by means of computer simulation using particles, whose motion is described by a Brownian dynamics (BD) algorithm. The potential advantages of our idea of a BD-based scheme alternative to those based on the Lamm equation, as already noted in other works (Walter et al. 2015; Thajudeen et al. 2017; Chaturvedi et al. 2017), is illustrated here for the case of samples of heterogeneous mass and density. The simplicity of the BD simulation makes it possible to extend the procedure easily to cases like those of a non-ideal solution, with physically or chemically interacting components, etc. In the present work, we have also initiated the development of computational tools, which will hopefully be useful for a variety of purposes in analytical ultracentrifugation.

The SimuSed programs, PrediSed and AnaSed, can be downloaded as executable files, along with their User’s Guides and sample files, from the website that hosts the HYDRO suite, at http://leonardo.inf.um.es/macromol.

## Electronic supplementary material

Below is the link to the electronic supplementary material.

Supplementary material 1 (PDF 51 kb)
