# Supplementary material for: Prediction and analysis of analytical ultracentrifugation experiments for heterogeneous macromolecules and nanoparticles based on Brownian dynamics simulation
Source: Eur Biophys J. 2018 Jul 20;47(7):845–54. doi: 10.1007/s00249-018-1322-2 (PMC6182663; doi:10.1007/s00249-018-1322-2)
Supplement: Supplementary file 1 — Supplementary material 1 (PDF 51 kb) [file 249_2018_1322_MOESM1_ESM.pdf]

Eur Biophys J manuscript No.
(will be inserted by the editor)

Prediction and analysis of analytical ultracentrifugation experiments for heterogeneous macromolecules and nanoparticles based on Brownian dynamics simulation

(Supplementary Material)

J. García de la Torre · J.G. Hernández Cifre · A.I. Díez Peña

Received: date / Accepted: date

Abstract This is a supplementary material where we present some numerical results that illustrate the uncertainty in the `PrediSed` results arising from the statistical noise of the Brownian dynamics simulation as well as the deviation of the `PrediSed` results from those of the `SEDFIT` reference.

Results are for the two-component system described in the main manuscript, with $s_1 = 1.91$ S, $M_1^{(b)} = 4250$ Da, $y_k = 0.60$, $s_2 = 7.9$ S, $M_2^{(b)} = 97350$ Da, $y_2 = 0.40$. Table A1 lists the specific parameters of the various cases considered. Cases labeled V/x and E/x correspond to typical sedimentation velocity (V) and sedimentation equilibrium (E), respectively. The cell bottom is at r_b= 7.2 cm in all cases.

J. García de la Torre
Department of Physical Chemistry, University of Murcia, 30071 Murcia, Spain
E-mail: jgt@um.es

J.G. Hernández Cifre
Department of Physical Chemistry, University of Murcia, 30071 Murcia, Spain
E-mail: jghc@um.es

A.I. Díez Peña
Department of Physical Chemistry, University of Murcia, 30071 Murcia, Spain
E-mail: anaisabel.diez@um.es

Table 1 Referred as Table A1

Case	ω/rpm	r_m/cm	r/cm	t/hours	`SEDFIT`
V/1	40000	5.8	6.435	3.92	0.537
V/2	40000	5.8	7.117	7.2	1.667
E/1	10000	6.5	6.531	100	0.381
E/2	10000	6.5	7.133	100	1.861
E/3	10000	6.5	6.900	100	0.612
E/4	10000	6.5	6.700	20	0.535

Table 2 Referred as Table A2

Case	N_{part}	N_r	N_s	Signal	Noise	Diff `SEDFIT`
V/1-1	10^5	50	101	0.545	0.017	0.008
V/1-2	10^7	50	101	0.5418	0.0016	0.0048
V/1-3	10^6	50	101	0.543	0.005	0.006
V/1-4	10^6	100	101	0.535	0.007	-0.001
V/1-5	10^6	200	101	0.541	0.013	0.004
V/1-6	10^6	800	101	0.531	0.016	-0.006
V/1-7	10^6	50	21	0.552	0.008	0.015
V/1-8	10^6	50	401	0.544	0.008	0.007
V/2-1	10^6	100	21	6.039	0.034	4.372
V/2-1	10^6	100	51	2.531	0.019	0.864
V/2-1	10^6	100	101	1.830	0.011	0.163
V/2-1	10^6	400	101	1.792	0.030	0.035
V/2-1	10^7	100	101	1.693	0.011	0.026

Numerical values are presented in Table A2 for the V cases and Table A3 for the E cases. Simulation parameters: number of molecules N_{part}, number of bins for radial position, N_r, and number of time steps N_s, are indicated. Signal is the `PrediSed` result for $z(r,t)/z_0$. Noise is the statistical uncertainty, obtained as the standard deviation, $\delta(z/z_0)$, of the results from 10 statistically independent `PrediSed` simulations, initiated with different seeds of the random number generator. This noise is found to be nearly the same throughout the cell during the whole sedimentation run. The reported values are averages over r and t. Inspection of the numerical results reveals that Noise $\propto \sqrt{N_r/N_{part}}$.

Diff `SEDFIT` is the deviation of the `PrediSed` results from the `SEDFIT` reference.

Table 3 Referred as Table A3

Case	N_{part}	N_r	N_s	Signal	Noise	Diff SEDFIT
E/1-1	10^6	100	101	0.414	0.009	0.033
E/1-2	10^6	100	201	0.401	0.010	0.020
E/1-3	10^6	100	401	0.398	0.009	0.017
E/1-4	10^7	100	201	0.405	0.003	0.022
E/1-5	10^7	1000	201	0.403	0.009	0.024
E/2-1	10^6	100	101	2.392	0.010	0.531
E/2-2	10^6	100	201	2.064	0.009	0.203
E/2-3	10^6	100	401	1.974	0.009	0.113
E/2-4	10^7	100	201	2.140	0.004	0.279
E/2-5	10^7	1000	201	2.107	0.010	0.246
E/3-1	10^6	100	101	0.615	0.009	0.003
E/3-2	10^6	100	201	0.613	0.008	-0.001
E/3-3	10^6	100	401	0.610	0.009	-0.002
E/3-4	10^7	100	201	0.615	0.003	0.003
E/3-5	10^7	1000	201	0.616	0.009	0.004
E/4-1	10^6	100	101	0.548	0.009	0.013
E/4-2	10^6	100	201	0.538	0.009	0.005
E/4-3	10^6	100	401	0.546	0.009	0.011
E/4-4	10^7	100	201	0.544	0.009	0.008
E/4-5	10^7	1000	201	0.547	0.009	0.012
